# AAPM‐RSS Medical Physics Practice Guideline 9.a. for SRS‐SBRT

**DOI:** 10.1002/acm2.12146

**Published:** 2017-08-08

**Authors:** Per H. Halvorsen, Eileen Cirino, Indra J. Das, Jeffrey A. Garrett, Jun Yang, Fang‐Fang Yin, Lynne A. Fairobent

**Affiliations:** ^1^ Radiation Oncology Lahey Health Burlington MA USA; ^2^ Radiation Oncology NYU Langone Medical Center New York NY USA; ^3^ Radiation Oncology Mississippi Baptist Medical Center Jackson MS USA; ^4^ Medical Physics Division Alliance Oncology Havertown PA USA; ^5^ Radiation Oncology Duke University Medical Center Durham NC USA; ^6^ Consultant Reston VA USA

## Abstract

The American Association of Physicists in Medicine (AAPM) is a nonprofit professional society whose primary purposes are to advance the science, education, and professional practice of medical physics. The AAPM has more than 8,000 members and is the principal organization of medical physicists in the United States.

The AAPM will periodically define new practice guidelines for medical physics practice to help advance the science of medical physics and to improve the quality of service to patients throughout the United States. Existing medical physics practice guidelines will be reviewed for revision or renewal, as appropriate, on their fifth anniversary or sooner.

Each medical physics practice guideline represents a policy statement by the AAPM, has undergone a thorough consensus process in which it has been subjected to extensive review, and requires the approval of the Professional Council. The medical physics practice guidelines recognize that the safe and effective use of diagnostic and therapeutic radiology requires specific training, skills, and techniques, as described in each document. Reproduction or modification of the published practice guidelines and technical standards by those entities not providing these services is not authorized.

The following terms are used in the AAPM practice guidelines:

- Must and Must Not: Used to indicate that adherence to the recommendation is considered necessary to conform to this practice guideline.
- Should and Should Not: Used to indicate a prudent practice to which exceptions may occasionally be made in appropriate circumstances.

Approved by AAPM Professional Council 3‐31‐2017 and Executive Committee 4‐4‐2017.

## 1 INTRODUCTION

The purpose of this Medical Physics Practice Guideline (MPPG) is to describe the minimum level of medical physics support deemed prudent for the practice of linear‐accelerator, photon‐based (linac) stereotactic radiosurgery (SRS), stereotactic (cranial) radiation therapy (SRT), and stereotactic body radiation therapy (SBRT) services. As SRS‐SBRT services are rapidly adopted into the community practice setting, this Guideline has been developed to provide appropriate minimum standards for such services.

### 1.A Scope

This MPPG's scope includes medical physics support for the entire treatment process including acceptance testing, commissioning, technical process development, treatment planning and delivery, and quality assurance related to linac‐based SRS, SRT, and SBRT, hereafter referred to as SRS‐SBRT. For ring‐mounted helical tomotherapy linac delivery systems, this document applies to SBRT only.^1^ This MPPG is not intended to address SRS‐SBRT procedures based on gamma ray and particle beam (proton or heavier) sources as well as linac‐magnetic resonance imaging (MRI) combination machines.

### 1.B Potential limitations and precautions

This MPPG describes the minimum level of medical physics support the American Association of Physicists in Medicine (AAPM) and the Radiosurgery Society (RSS) deem prudent for the aforementioned scope. This document does not constitute a policy and procedure or standard operating procedure for a specific clinic — that is the professional responsibility of the clinic's Qualified Medical Physicist^1^ through an active collaboration with the clinic's Medical Director and other clinical team members.

### 1.C Definitions

1. End‐to‐end (E2E) testing — a methodology used to test whether the flow of an application is performing as designed from start to finish. The purpose of carrying out E2E tests in radiation oncology is to identify system dependencies, to ensure that the intended information is correctly passed between various system components, to verify that clinical team members understand their tasks, and to assess overall treatment process accuracy. All aspects of the treatment process should be considered, including immobilization, simulation, respiratory‐related motion management, treatment planning, and treatment delivery using a clinically relevant image guidance method. Each step in the E2E testing should be performed by the staff member who will perform the step when the program is clinically implemented.
2. Medical dosimetrist — a person other than a radiation oncologist or medical physicist who participates in, performs, and/or assists in the procedures required to develop a radiotherapy treatment plan with related treatment delivery parameters, working under the supervision of a radiation oncologist and qualified medical physicist (QMP).
3. Quality Assurance (QA) — as defined in the AAPM Task Group 100 report:^2^ “QA confirms the desired level of quality by demonstrating that the quality goals for a task or parameter are met.” In the context of this document, QA refers to the programmatic approach to ensuring quality and safety in SRS‐SBRT treatments.
4. Qualified Medical Physicist (QMP) — as defined by AAPM Professional Policy 1.^1^ For this practice guideline, the applicable subfield is therapeutic medical physics.
5. Quality Control (QC) — as defined in the AAPM Task Group 100 report:^2^ “QC encompasses procedures that force the desirable level of quality by evaluating the current status of a treatment parameter, comparing the parameter with the desired value, and acting on the difference to achieve the goal.” In the context of this document, QC refers to specific tests performed as described in the QA program.
6. Stereotactic Radiosurgery (SRS) — as defined in the American College of Radiology–the American Society for Radiation Oncology (ACR‐ASTRO) Practice Parameter for the Performance of Stereotactic Radiosurgery:^3^ “For the purpose of this document, SRS is strictly defined as radiation therapy delivered via stereotactic guidance with approximately 1 mm targeting accuracy to intracranial targets in 1–5 fractions.”
7. Stereotactic Body Radiation Therapy (SBRT) — as defined in the ACR‐ASTRO Practice Parameter for the Performance of Stereotactic Body Radiation Therapy^4^: “Although treatment of intracranial sites may be understood conceptually as a form of SBRT, for the purpose of this document, SBRT is strictly defined as radiation therapy delivered via stereotactic guidance with high levels of targeting accuracy to extracranial targets.”

## 2 STAFF QUALIFICATIONS AND RESPONSIBILITIES

Each member of the SRS‐SBRT team must be appropriately trained, and each team member's responsibilities in the SRS‐SBRT process must be clearly defined to ensure a consistently safe and accurate treatment delivery. (Training is addressed in more detail in Section 4.B.2 of this document.)

### 2.A Supervision level

This document follows supervision levels defined in AAPM Professional Policy 18.^5^ For the delivery of all radiation therapy services, the two responsible professionals are the radiation oncologist and QMP. All other team members work under the supervision of these professionals — clinical procedures supervised by the radiation oncologist and technical procedures supervised by the QMP.

1. General Supervision: The procedure is performed under the professional's overall direction and control but the professional's presence is not required during the performance of the procedure. Under General Supervision, the training of the personnel who performs the procedure and the maintenance of personnel competence are the continuing responsibility of the professional.
2. Direct Supervision: The professional must exercise General Supervision and be present in the facility and immediately available to furnish assistance and direction throughout the performance of the procedure.
3. Personal Supervision: The professional must exercise General Supervision and be present in the room during the performance of the procedure.

### 2.B Medical physicist

#### 2.B.1 Qualifications

The medical physicist with responsibility for the SRS‐SBRT program should meet the AAPM definition of a QMP^1^ for therapeutic medical physics. Appropriately trained medical physicists who do not meet the definition of a QMP work under the supervision of a QMP. All medical physicists supporting the SRS‐SBRT program should have specific training in SRS‐SBRT prior to supervising patient‐specific procedures, including review of planning and treatment delivery procedures, equipment‐specific QA, and patient‐specific QA.

#### 2.B.2 Responsibilities

1. As stated in the ACR‐ASTRO Practice Parameter for SRS:^3^ “The medical physicist is responsible for the technical aspects of radiosurgery and must be available for consultation throughout the entire procedure: imaging, treatment planning, and dose delivery.” The ACR‐ASTRO Practice Parameter for SBRT^4^ describes the medical physicist's responsibility similarly for SBRT.
2. Perform acceptance testing and commissioning of the SRS‐SBRT system, including validation of the treatment planning system accuracy with small fields and tissue heterogeneities (if relevant to the scope of SRS‐SBRT services offered), accuracy of targeting through end‐to‐end (E2E) testing, and quality and precision of the image guidance system.
3. Implement and manage a QA program to ensure proper ongoing performance of the treatment delivery unit, immobilization and simulation devices, image guidance system, and treatment planning system.
4. Work with other team members to develop standard operating procedures (SOPs) for major steps through the entire treatment process.
5. Establish a comprehensive safety checklist to act as a guide for the entire treatment process, and determine appropriate methods for the clinic's quality assurance committee to monitor the SRS‐SBRT program.
6. Facilitate and manage the clinic's participation in an incident learning system to ensure a transparent, structured evaluation of all “near miss” and actual deviations in the planning and treatment delivery process.
7. Perform or supervise the dosimetric treatment planning process, providing supervision levels as appropriate to each task (e.g., direct supervision at the initial and final phases of the treatment planning process).
8. Review the final treatment plan for accuracy and deliverability, consulting with the radiation oncologist to ensure that both professionals are confident of the acceptability of the chosen treatment plan.
9. Validate the chosen treatment delivery parameters via an independent dose calculation. When deemed appropriate, a phantom measurement or treatment delivery “dry run” may also be performed.
10. For the first treatment session, a QMP with relevant SRS‐SBRT training must provide personal supervision of the entire session.^2^ For any subsequent treatment sessions, direct supervision must be provided by either a QMP or a medical physicist who was present during the initial treatment session.

### 2.C Radiation oncologist

#### 2.C.1 Qualifications

The radiation oncologist should be certified in Radiation Oncology or Therapeutic Radiology by the American Board of Radiology and have completed specific training in SRS‐SBRT as stated in the ACR‐ASTRO Practice Parameter for SRS^3^ and the ACR‐ASTRO Practice Parameter for SBRT^4^ prior to commencing SRS‐SBRT services.

#### 2.C.2 Responsibilities

As stated in the ACR‐ASTRO Practice Parameter for SRS^3^ and the ACR‐ASTRO Practice Parameter for SBRT.^4^

### 2.D Medical dosimetrist

#### 2.D.1 Qualifications

A dosimetrist providing SRS‐SBRT treatment planning services should be certified by the Medical Dosimetry Certification Board, have specific training in SRS‐SBRT planning prior to performing patient‐specific procedures, and be supervised by a radiation oncologist and QMP. The QMP with responsibility for the SRS‐SBRT program is responsible for determining the competency of each dosimetrist to provide SRS‐SBRT treatment planning services. Note: A medical physicist with appropriate training in SRS‐SBRT planning may perform the activities listed under Section 2.D.2 below.

#### 2.D.2 Responsibilities

1. Participate in simulation sessions as needed to be aware of and provide suggestions related to selection of patient immobilization and likely beam paths, motion effects on target coverage, and other dosimetric considerations.
2. Under the radiation oncologist's and QMP's supervision, delineate normal tissue volumes, assess the target volume for contiguity and proximity to dose‐limiting normal tissues, review findings with the radiation oncologist, and generate treatment plan(s) in accordance with the patient‐specific dosimetric objectives. The QMP supervises the technical aspects of the treatment planning process and consults with the radiation oncologist regarding any additional considerations such as prior treatment, motion management, and implanted medical devices.
3. Upon approval of a treatment plan by the radiation oncologist, document the chosen treatment technique and if necessary, export all relevant delivery parameters to the electronic treatment management and image guidance system(s). Coordinate with the QMP to ensure that appropriate plan review and quality assurance are completed prior to initiation of treatment.
4. Ensure that all aspects of the chosen treatment technique are clearly conveyed to the therapist team. For unusual or complex aspects of a patient's treatment technique, communicate directly with the therapists to ensure that they are aware.

### 2.E Radiation therapist

#### 2.E.1 Qualifications

All radiation therapists should hold an active certification in radiation therapy by the American Registry of Radiologic Technologists, maintain licensure as required by the state, and have specific training in the clinic's SRS‐SBRT procedures prior to performing such SRS‐SBRT procedures.

#### 2.E.2 Responsibilities

For each simulation session, prepare immobilization devices, position patient, and acquire images for treatment planning in accordance with the clinic's SRS‐SBRT procedure and the patient‐specific instructions. Document treatment setup parameters after the radiation oncologist has approved the positioning, images, and target localization.

If the QMP has delegated certain daily QC tasks, perform the relevant QC tests under the QMP's supervision following the procedure established by the QMP.

For each treatment session, prepare the treatment room for the SRS‐SBRT procedure in accordance with the clinic's procedure and the patient‐specific instructions, position the patient and localize the treatment isocenter, and operate the treatment unit after the radiation oncologist and QMP have approved the clinical and technical aspects of the treatment delivery.

## 3 RESOURCES

Because of the high dose per fraction and the critical importance of targeting accuracy, SRS‐SBRT services require a strong commitment by the radiation oncology program and the facility to provide the appropriate resources. Both the clinical team and the institution's administration must understand their roles and commitments in this regard prior to implementing SRS‐SBRT services. In this context, “resources” refers to appropriate staffing and coverage, appropriate equipment to support the treatment process, appropriate instruments for QC, clear operating procedures with delineation of duties and appropriate time intervals for all staff to safely perform their work, and a safety culture rooted in transparency and process analysis.

An institution should not offer SRS‐SBRT services unless it can provide the following resources and supports the following programmatic imperatives.

### 3.A Staffing and coverage

1. Adequate medical physicist staffing to ensure that a QMP with appropriate SRS‐SBRT training is available to review QC results and consult with the clinical team on patient‐specific aspects of treatment planning and delivery and to provide personal supervision for the first treatment session of every SRS‐SBRT treatment course and as necessary for portions of all subsequent treatment fractions.
2. Adequate radiation oncologist staffing to ensure that a radiation oncologist with appropriate SRS‐SBRT training is available for direct or personal supervision (as described in the department's SOPs) of the simulation, treatment planning, and treatment delivery of every SRS‐SBRT treatment course.
3. Adequate staffing to ensure that a dosimetrist or medical physicist with appropriate SRS‐SBRT training can devote the time necessary to develop a treatment plan with comprehensive review of all technical aspects such as prior treatment, respiratory motion of target and adjacent organs, multimodality image registration, and potential limitations in treatment delivery.
4. Adequate therapist staffing to ensure that at least two certified radiation therapists are present for every treatment session, with at least one therapist who is appropriately trained in the SRS‐SBRT treatment technique being used.

### 3.B Instrumentation

1. Redundant radiation detectors suitable for small fields.
2. Reference‐grade electrometer suitable for low‐signal readings.
3. Appropriate E2E phantoms for the scope of SRS‐SBRT services offered, available for use on site in a timely manner (not to exceed 72 hr).
4. QC device to perform a radiation isocentricity beam alignment verification.

### 3.C Simulation, planning, and treatment resources

1. Appropriate devices for patient setup and immobilization.
2. Appropriate devices for proper motion management.
3. Computerized treatment verification system.
4. Digital access to MRI and positron emission tomography (PET) image data.
5. Capability to characterize and quantify internal anatomical changes with respiratory motion (for thoracic and abdominal SBRT services).
6. Multimodality image fusion capability.
7. Capability to calculate, display, and evaluate composite dose for patients who have received prior radiation therapy.
8. Linac‐based treatment delivery system with appropriate mechanical accuracy, field‐aperture size, and resolution for small‐target conformality, and image‐guidance devices for target localization and verification including motion management technology relevant to the scope of SRS‐SBRT services to be offered.^6^, ^7^

### 3.D Administrative support

1. Commitment to support the delineation of duties, procedure‐specific QA, and staff authority required for safe delivery of SRS‐SBRT services, as defined in SOPs developed by the institution's QMP and medical director of radiation oncology consistent with the institution's credentialing process.
2. Commitment to facilitate and pay for independent peer review of the SRS‐SBRT program and on‐site proctoring of the first SRS‐SBRT treatment(s) if the clinical team does not have relevant prior experience with the SRS‐SBRT service being implemented at the center.^8^
3. Commitment to support ongoing training for members of the SRS‐SBRT team as deemed necessary by the medical director and QMP. Such training may be necessitated by staff turnover, changes in staff roles, or low SRS‐SBRT procedure volume resulting in infrequent staff experience.^8^
4. Robust preventive maintenance and field service support arrangements for the key simulation, planning, and treatment delivery systems.

## 4 ACCEPTANCE TESTING AND COMMISSIONING

### 4.A Acceptance testing

The QMP must be involved with the process of facility design, equipment selection and specifications, and provide direct supervision during the acceptance testing process.^9^ Customer acceptance test procedures are intended to ensure that the equipment satisfies the performance requirements stated in the purchase agreement, including that the equipment is safe to operate. In some cases, measurements completed as part of the acceptance procedures may also serve as components in establishing the routine quality assurance program. The vendor must demonstrate acceptable system performance.

### 4.B Commissioning

To determine the scope of SRS‐SBRT commissioning, the QMP must understand the scope of procedures/services to be offered. Commissioning encompasses the overall process of validating the planning and delivery system for the services to be offered, and developing appropriate QC and technical procedures to support these services. The scope of commissioning must therefore be commensurate with the scope of clinical services to be offered.

#### 4.B.1 Equipment commissioning

Commissioning of a linac‐based treatment delivery system is performed after acceptance testing. Commissioning tests should be developed by the institution's medical physics team to explore in detail every aspect of the system with the goal of developing a comprehensive baseline characterization of the performance of the system, identifying any limitations relative to clinical use, and developing procedures for QA and clinical use.^10^, ^11^ A variety of task group reports are referenced in this document to provide guidance on best practice for performing commissioning and QA of delivery devices. However, SRS‐SBRT intent requires special consideration.

Each SRS‐SBRT system is highly specialized with fixed cones and/or multileaf collimators (MLCs). Specific validation should be considered based on manufacturer recommendations and the determined scope of the practice. Commissioning of such systems includes, but is not limited to, a safety and geometric accuracy evaluation of the treatment and imaging components, comprehensive small‐field data measurement with appropriate stereotactic detectors and careful equipment setup, evaluation of treatment planning system capabilities including multimodality image processing and calculation accuracy for small fields, and the development of a comprehensive QA program for each of the following critical components:

1. Treatment delivery machine
2. Immobilization devices^12^
3. Ancillary systems for imaging^13^ and motion management
4. Treatment planning systems^14^

##### Special consideration: Small‐field measurements

Small‐field dosimetry as used in SRS‐SBRT is challenging due to many factors including source size, detector size, and response.^15^ As a generalization, even micro‐ion‐chambers are large relative to the field sizes used in SRS‐SBRT due to violation of cavity theory.^16^, ^17^ Generalized approaches to the lack of lateral equilibrium and violation of cavity theory have been addressed in the literature.^18^, ^19^, ^20^, ^21^ Newer solid‐state microdetectors have become available such as diode, plastic scintillators, and synthetic microdiamonds that have shown appropriate characteristics for small‐field dosimetry. Evaluations of many commercially available detectors have been published with correction factors for small‐field dosimetry.^22^ A practical measurement methodology for validating small‐field beam data using multiple detectors has also been reported.^23^ A newly published code of practice from the International Atomic Energy Agency^24^ is also a useful guideline. An important characteristic of any detector used for commissioning is that the detector's active area be of a small size compared to the field size range to be characterized. A daisy‐chain method is recommended, using two independent detectors suitable for measuring small fields.^14^, ^22^

Upon completion of beam data measurements, key data points (such as percent depth dose at 10 cm depth and output factors for field sizes ≤2.0 cm) should be compared to other machines of identical design, whether in the same institution or from other centers, to guard against gross errors which could arise from inappropriate detector selection or misaligned equipment setup.

##### Immobilization equipment

Immobilization equipment should be evaluated for its effectiveness in targeting accuracy and precision (e.g., through analysis of shifts after pretreatment imaging for a representative sample of patients for each treatment site), and should be evaluated for its beam attenuation and surface dose characteristics.^12^ The effect on surface dose should be clearly articulated to the clinical team prior to implementation of the clinical service.

##### Treatment planning system

Commissioning of the treatment planning system's dose model should include all aspects described in AAPM Medical Physics Practice Guideline 5,^14^ with additional validation tests as appropriate for the specific SRS‐SBRT delivery technology and scope of clinical services such as evaluation of multimodality image fusion accuracy, validation of clinically relevant small‐field dose calculations (using cone, Iris^TM^, or MLC fields if in scope), calculation accuracy for couch attenuation and effect on surface dose,^12^ and heterogeneity corrections. Note that pencil‐beam dose algorithms are not appropriate for extracranial SRS‐SBRT applications where the beam paths traverse significant tissue heterogeneities, such as for lung, dome of liver, and nasopharynx treatment sites.^25^, ^26^

##### Motion management

If the planned scope of clinical services includes treatments affected by respiratory motion, the entire treatment chain (CT simulation, treatment planning, and treatment delivery) should be assessed with E2E testing using a dynamic phantom setup with clinically relevant motion parameters (amplitude, cycle time). The tests should include assessment of spatial targeting accuracy and measurement of delivered target dose.

##### Independent review

All new SRS‐SBRT programs should have independent validation of the beam model and machine calibration prior to initiation of the clinical service. This can be accomplished through E2E phantom tests from the Imaging and Radiation Oncology Core,^25^ or through an independent physicist's on‐site review.^27^

##### Commissioning report

The scope of commissioning work and key results should be summarized in a written commissioning report. The report should clearly identify known limitations in the delivery chain, limits for clinical implementation (e.g., minimum field size), and baseline data to support the equipment QC program. If the full commissioning report is not completed prior to initiation of the clinical service, an Executive Summary describing the known limitations and limits for clinical implementation must be prepared and shared with the clinical team prior to initiation of the clinical service.

#### 4.B.2 Process commissioning and clinical implementation

Clinical implementation of a stereotactic program requires agreement within the clinical team on the scope and clinical goals of the program. Development and validation of the technical process to be followed for delivering a clinical SRS‐SBRT service may be regarded as process commissioning, and should be completed prior to the first patient treatment. The SOPs for each anatomical site to be treated should be developed in collaboration with the clinical team. That team includes the radiation oncologist, the QMP, the medical dosimetrist, the radiation therapist, and often the radiation oncology nurse. There are several references available including AAPM task group reports,^10^, ^28^ ACR‐ASTRO Practice Parameters,^3^, ^4^ and recent AAPM Medical Physics Practice Guidelines.^14^, ^29^, ^30^ Each of these references should be reviewed to develop an overall understanding of the scope of a stereotactic program implementation.

Specific clinical implementation guidance is found in Section VII of the AAPM TG 101 report.^10^ These components described in the TG 101 report are also applicable to SRS procedures. The following section is consistent with the TG 101 recommendations, providing additional details deemed relevant to a clinical SRS‐SBRT program.

##### Standard operating procedures

Site‐specific SOPs should address the components essential to the patient review, simulation, planning, treatment, and follow‐up (see the Appendix for a sample SOP document). Patient safety should be the primary consideration when developing any SOP.

1. Safety
  - The roles and responsibilities of each member of the clinical team should be clearly described in the SOP document. See section 2 of this Practice Guideline for additional information, and the Appendix for a sample SOP document.
  - Mechanical tolerances will be established during commissioning and should be well documented. Additional tolerances for clinical operation should be considered for each SRS‐SBRT service, and should be clearly defined in the SOP document.
  - The SOP should establish certain process expectations for safe implementation such as appropriate time intervals from simulation to treatment with critical points along the path allowing for reconsideration or rescheduling.
  - Every team member has the right and responsibility to halt a case and/or a particular procedure based on safety imperatives.
2. Patient selection
  - Patient selection criteria should initially be determined using data available from clinical protocols or published guidelines. Maximum target size should be documented along with standard prescription dose and fractionation schemes.
  - Where possible, a multidisciplinary review or a peer review of proposed cases should be completed prior to simulation. If the patient is enrolled in a clinical trial, the rules and guidelines of the clinical trial must be followed.
3. Simulation
  - Reproducible immobilization techniques should be developed for each treatment site.
  - The reference imaging study to be used for treatment planning should cover the target and all relevant organs at risk. A typical scan length should extend at least 10 cm beyond the treatment field borders. For non‐coplanar treatment techniques, the scan length may need to be further extended to adequately model the beam paths and resultant scatter dose^10^ and extend beyond the entrance path and clinically relevant exit path of every beam.
  - For SBRT applications, tomographic slice thickness of 1–3 mm should be used. For SRS applications, slice thickness should not exceed 1.25 mm and scan field of view should be optimized for maximum in‐plane spatial resolution while including all necessary anatomy and immobilization hardware in the field of view.
  - Respiratory motion management should be considered in thoracic and abdominal sites. At least one of the five categories of motion management as described in the AAPM Task Group 76 report^31^ should be implemented, with a QA program consistent with the TG‐76 recommendations. The TG‐76 report includes a flowchart for assessing and managing respiratory motion.
4. Treatment planning
  - The treatment planning system must have the capability of accurately calculating the predicted dose for the scope of SRS‐SBRT services to be offered.^10^, ^14^
  - Each treatment site should have a defined list of critical structures to evaluate and stereotactic fractionation based tolerances should be defined based on clinical protocol data or peer‐reviewed literature.^32^ The QMP should ensure that the radiation oncologists are aware of the delivery system's tolerances relative to the planning target volume and organs at‐risk avoidance margins. The planning target volume margins should be clearly documented.
  - Image fusion requirements for target definition should be defined and target margins should be clearly described. Target margins should be based on data from current literature along with knowledge of the limitations of in‐house localization capabilities.
  - Planning strategies and techniques should be described for each treatment site, such as conformal arcs, intensity‐modulated radiation therapy, and volumetric‐modulated arc therapy. These technique definitions should include clinical limitations based on the findings from commissioning. If noncoplanar techniques are included, potential collision should be considered in determining overall beam configuration.
  - In cases of re‐irradiation, the cumulative dose should be evaluated by the treating physician. A description of the method used and the outcome of the evaluation should be documented.
  - The use of an isotropic calculation grid size of 2 mm or finer is recommended. The use of a grid size >3 mm is discouraged.^10^ For very small targets, a 1 mm calculation grid size may be necessary.
  - Target dose coverage, dose fall‐off beyond the target, dose conformity metrics, and compliance with critical structure dose objectives 32 should be clearly reported and signed by the radiation oncologist to confirm that the chosen treatment technique is clinically acceptable.
  - An independent dose calculation check must be performed prior to treatment.
5. Treatment delivery
  - A clearly defined pretreatment QA check should be performed and may depend on the technique used^33^ (e.g., frameless cranial, frame‐based cranial, cone‐based SRS or SBRT). This should include a collision check where the potential for collision exists.
  - The SOP should clearly describe the professional supervision requirements for each SRS‐SBRT treatment type.^3^, ^4^, ^5^, ^10^, ^28^
  - The SOP should clearly describe the image guidance method to be used, including target anatomy, critical organ avoidance, and localization tolerance. Pretreatment verification of target localization should always be performed; the criteria for intratreatment image guidance should be clearly described.^34^
  - If motion management is used, the SOP should clearly describe the process, tolerances, and professional supervision.
6. Patient follow‐up The SOP should clearly describe the follow‐up schedule and clinical tests for each treatment site. “There should be follow‐up of all patients treated, and appropriate records should be maintained to determine local control, survival, and normal tissue injury.”^3^, ^4^
7. Checklists Effective checklists support human thinking, allow constructive team member interactions, and facilitate a systematic care delivery by reducing process variability. The AAPM Medical Physics Practice Guideline on development and implementation of safety checklists^30^ should be followed in developing treatment‐specific checklists.

##### Training

Training should address the need for initial as well as ongoing training and should be supported by a system of documentation and checklists to ensure that all team members are competent to support the clinical service.^8^

1. Training venues
  - Vendor training — When possible, a core team should participate in all available vendor training on relevant hardware and software including off‐site training, onsite training, and case observation.
  - Nonvendor training — Attendance at structured courses and/or “shadowing” procedures at a facility with a mature SRS/SRT/SBRT service should be considered. This should include a review of the SOP for the SRS‐SBRT services to be implemented, including equipment‐specific and patient‐specific QA procedures.
  - If the principal professionals responsible for the SRS‐SBRT service do not have direct prior experience with the services to be offered, the facility must arrange for on‐site review and proctoring of the first clinical procedure by professionals with experience relevant to the new service.
2. Ongoing competency
  - The competency checklist should be reviewed periodically (at least annually) and updated as the program evolves.^8^
3. Documentation
  - Written standard operating procedures must be developed, and reviewed by all participating staff.
  - All training should be documented.
  - A checklist of relevant competencies should be developed and the checklist completed prior to program implementation.

##### End‐to‐End (E2E) testing

To assess the clinical team's readiness and to validate the SOP, the team should conduct “dry runs” of the entire process, observe and take notes, edit the SOP as needed, and repeat the E2E testing until the process is clear to all participants. The pre‐implementation E2E tests and findings should be described in the commissioning report.

Each step in the E2E testing should be performed by the staff member who will perform the step when the program is clinically implemented. E2E process “dry runs” should be performed for each category of SRS‐SBRT service, and when a key aspect of the process is changed.

When developing the E2E tests, all aspects of the treatment process should be considered, including immobilization, simulation, respiratory management, treatment planning, and treatment delivery using a clinically relevant image guidance method.

## 5 QUALITY ASSURANCE

### 5.A Introduction

A comprehensive QA program for SRS‐SBRT is critical to ensure the correct dose is delivered to the target, given the very small target volumes and rapid dose fall‐off associated with SRS‐SBRT. QA processes and procedures related to SRS‐SBRT should be designed to cover the following aspects of the SRS‐SBRT program: equipment‐specific QA, patient‐specific QA, and procedure‐specific QA. Safety and QA recommendations have been extensively described in several publications.^3^, ^4^, ^10^, ^33^

When equipment performance is found to be out of tolerance, the affected module(s) of the delivery system should be promptly adjusted, and the QMP should verify proper performance before clinical SRS‐SBRT services resume. In the event of a significant service interruption, the QMP should coordinate closely with treating physicians to evaluate the impact on patients’ treatment schedules given the importance of completing SRS‐SBRT treatment courses in a short overall time interval (generally 14 days or less).^35^ Patient safety should be the primary consideration in determining when to resume clinical services.

### 5.B Minimum equipment‐specific QA

The AAPM has published task group reports with recommendations for QA related to SRS‐SBRT. TG‐142 describes the linear accelerator QA for both conventional radiation therapy procedures and for SRS‐SBRT procedures.^36^ MPPG 2.a provides recommendations for commissioning and quality assurance of X‐ray–based image‐guided radiotherapy systems.^29^ TG‐135 provides specific guidance for QA of robotic radiosurgery systems,^28^ and TG‐148 provides specific guidance for QA of helical tomotherapy systems.^37^ MPPG 5.a provides minimum QA recommendations for treatment planning system dose algorithms.^14^ The baseline performance values for routine equipment QA (daily, monthly, and annual QA) should be established during machine commissioning and initial calibration. The SRS‐SBRT relevant QA tests, frequencies, and tolerances are summarized in Tables 1–3 for C‐arm linac, robotic linac, and ring‐mounted helical tomotherapy systems, respectively. In addition, each CT scanner used for treatment planning should be evaluated at least annually to confirm geometric accuracy^13^ and constancy of the CT number to density curve.^14^

**Table 1:** Minimum SRS‐SBRT relevant equipment QA and tolerances for C‐arm linac systems

Frequency	Test	Tolerance
Daily	Laser localization — only if using SRS techniques relying on lasers for target localization (e.g., frame‐based SRS without X‐ray IGRT)	1 mm
	Collimator size indicator for clinically relevant aperture	2 mm total
	Radiation isocentricity test (limited gantry and couch positions) — maximum deviation in center of target object relative to each projection's beam central axis	1.0 mm SRS, 1.5 mm SBRT
	IGRT positioning/repositioning	1 mm SRS, 2 mm SBRT
	Imaging subsystem interlocks	Functional
	Stereotactic interlocks — cone size, backup jaws	Functional
	Accelerator output constancy	±3%
Monthly	Radiation isocentricity test — covering complete range of gantry, couch, collimator positions used clinically — *maximum deviation in center of target object relative to each projection's beam central axis* *Note: If both MLC and fixed conical collimators are used, both must be evaluated at least monthly	1.0 mm SRS, 1.5 mm SBRT
	Treatment couch position indicators: relative over the maximum clinical range	1 mm/0.5°
	Output constancy at relevant dose rates	2%
Annually	SRS arc rotation mode (if used clinically)	1 MU, 1°
	MU linearity (≥5 MU to highest MU used clinically)	±2%
	Accelerator output	±1.5%
	Coincidence of radiation and mechanical isocenter	±1.0 mm maximum 3‐D displacement from center of target object
	Verification of small‐field beam data — relative output factors for cones and/or MLC	±2% from baseline for >1.0 cm apertures, ±5% from baseline for ≤1.0 cm apertures
	E2E localization assessment “hidden target test” using SRS frame and/or IGRT system	1.0 mm
	E2E dosimetric evaluation using SRS frame and/or IGRT system	±5% measured vs. calculated

Tolerances are absolute accuracy, not variation from baseline, unless otherwise stated.

**Table 2:** Minimum equipment QA and tolerances for robotic linac systems

Frequency	Test	Tolerance
Daily* *On days of clinical use	Head laser alignment check	1.0 mm
	Safety interlocks	Functional
	Automatic QA (AQA) test* *If the clinic has both fixed cones and Iris ^*TM*^ *collimator, the AQA test should alternate between fixed cones and Iris* ^*TM*^ *, with each system tested at least weekly*	Total targeting ≤1.0 mm from baseline, not exceeding manufacturer's specification
	Accelerator output constancy	±3%
Monthly	Energy constancy	±2%
	Beam symmetry, relative	±3% for 40 mm field, ±4% for 60 mm field
	Accelerator output constancy	±2%
	Imager alignment	1 mm or center pixels ±2 pixels
	Iris^TM^ field size spot check (3 or more field sizes ≥10 mm)	0.5 mm
	Picket fence for MLC (if applicable)	Visual check
Quarterly	E2E localization assessment (Each tracking mode used clinically)	1.0 mm static target, 1.5 mm motion tracking
Annually	Emergency Power Off (EPO) button, safety interlocks	Functional
	Accelerator output	±2.0%
	MU linearity (>10 MU to highest MU used clinically)	±2%
	Path verification	≤0.5 mm maximum per node, ≤0.3 mm average
	Imager kVp accuracy, mA station exposure linearity, isopost alignment with center pixel	±10%, ±20%, and 1 mm, respectively
	Beam laser and radiation beam alignment for cone, Iris^TM^, and MLC	0.5 mm from baseline
	AQA baseline	Recheck AQA baseline
	Beam data verification — Relative output factors for cones, Iris^TM^, and/or MLC covering the range used clinically	±2% from baseline for >1.0 cm apertures, ±5% from baseline for ≤1.0 cm apertures

**Table 3:** Minimum SBRT relevant equipment QA and tolerances for helical tomotherapy systems

Frequency	Test	Tolerance
Daily	Red laser initialization (congruence with green laser)	1 mm
	Image/laser coordinate coincidence	1 mm
	Image registration/alignment	1 mm
	Accelerator output constancy (rotational or static)	±3%
Monthly	Transverse beam profile	1% average difference in field core
	Longitudinal beam profile (each slice width)	1% of slice width FWHM
	Output constancy and rotational output variation	±2%
	Beam quality constancy	±1% PDD_10_ or TMR^20^_10_
	Red and green laser alignment	1 mm
	Couch positioning accuracy	1 mm
	CT dimensional accuracy	1 mm
Annually	Couch speed uniformity	±2% dose nonuniformity
	Couch translation per gantry rotation	1 mm per 5 cm
	Accelerator output	±1.5%
	Beam quality (each slice width)	±1% PDD_10_ or TMR^20^_10_
	Verification of small‐field beam data	±2% from baseline for >1.0 cm apertures, ±5% from baseline for ≤1.0 cm apertures
	CT imaging — treatment — laser coordinate coincidence	1.0 mm
	E2E localization assessment “hidden target test”	1.0 mm
	E2E dosimetric evaluation	±5% measured vs. calculated

SRS is not included in the scope of this document for helical tomotherapy.

Tolerances are absolute accuracy, not variation from baseline, unless otherwise stated.

Note: Many tests described in the aforementioned AAPM publications are important for characterizing the system performance regardless of the scope of clinical use; the equipment‐specific QA in Tables 1 through 3 are those deemed most directly relevant to the SRS‐SBRT service. The QMP responsible for the clinic's QA program should consider all recommendations in the aforementioned AAPM publications for their relevance to the clinic's overall scope of services.

### 5.C Patient‐specific QA

#### 5.C.1 Overview

Compared with conventionally fractionated radiotherapy, the target volume in SRS and SBRT is much smaller, the dose heterogeneity is higher, and the dose falls off faster in tissue. The term “Patient‐specific QA (PSQA)” for SRS and SBRT, in the context of this Practice Guideline, refers to verifying that the approved treatment plan can be accurately delivered.

#### 5.C.2 Scope of PSQA

Patient‐specific QA should include verification of patient setup/immobilization, independent check of the approved treatment plan and associated treatment delivery parameters, dose delivery measurements when appropriate, chart rounds and/or peer review, and a dry‐run of the approved treatment plan to check for potential collision. If fixed conical collimators are used, dose delivery measurements are prudent to verify the integrity of treatment, but are not essential as the measured dosimetric characteristics (profile, output, Tissue Phantom Ratio, etc.) are directly applied to the dose calculation. When the MLC is applied to modulate the dose, dose delivery measurements should be performed prior to treatment to verify the absolute dose to the reference point (usually isocenter).

#### 5.C.3 Instrumentation for PSQA

The QMP determines the instrumentation appropriate to the SRS‐SBRT technique to be verified. Common instrumentation includes radiochromic film, small‐volume ion chamber (for relatively larger treatment fields), diode detector, portal imaging device calibrated for dose response, detector arrays, and, less commonly, polymer gel dosimetry. The institution must provide appropriate instrumentation to conduct PSQA as deemed necessary by the QMP. The clinical service should not be initiated if appropriate instrumentation is not available for the QMP's use.

### 5.D Procedure‐specific QA

Procedure‐specific QA addresses issues related to operational tasks, such as checking whether:

1. The workflows to perform SRS‐SBRT as defined in the SOP documents are consistently followed.
2. Staffing level is appropriate.
3. Staff training and continuous training are available and appropriate.
4. Proper follow‐up actions are taken for any actual and/or potential (“near miss”) treatment incidents.

As described in the Clinical Implementation section above, each facility should have SOP documents defining the workflow of each SRS‐SBRT service. These documents should be reviewed and updated regularly, with at least an annual frequency of review.

Staffing levels, training, and competency assessments are critical for a successful SRS‐SBRT program. Team members without prior relevant SRS‐SBRT experience should perform a minimum of five procedures working under the supervision of an experienced expert for each SRS‐SBRT service.

Ongoing competency assessment is necessary given the rapid evolution of technology and treatment methods for SRS‐SBRT. These activities should be properly documented.

### 5.E QA program supervision

The QA program should be designed by a QMP who has specific training in SRS‐SBRT, and should be reviewed by another QMP with SRS‐SBRT experience. The daily QA procedure can be performed by a physicist or radiation therapist and be reviewed by the QMP prior to any SRS‐SBRT treatment. Other routine QA or patient‐specific QA may be performed by an appropriately trained medical physicist, and reviewed and co‐signed by the QMP.

### 5.F QA program review

When the SRS‐SBRT program is in its initial phase, the QA program should be reviewed bi‐annually as the clinical practice and utilization evolves. The frequency can be reduced to annual reviews once the clinical practice and utilization stabilizes.

## 6 CONCLUSIONS

For the delivery of SRS‐SBRT services, the two responsible professionals are the radiation oncologist and QMP. All other team members work under the supervision of these professionals — clinical procedures supervised by the radiation oncologist and technical procedures supervised by the QMP. The provision of SRS‐SBRT services should follow a structured approach with clearly defined roles, responsibilities, procedures, and action levels. The clinic's QMP develops SOPs for SRS‐SBRT through an active collaboration with the clinic's medical director.

The resources and programmatic components described in Section 3 are imperative to safe implementation of SRS‐SBRT services.

The scope of commissioning work and key results should be summarized in a written commissioning report. The report should clearly identify known limitations in the delivery chain, limits for clinical implementation (e.g., minimum field size), and baseline data to support the equipment QC program. Any relevant limitations must be clearly communicated to the clinical team prior to program implementation.

All new SRS‐SBRT programs should have independent validation of the beam model and machine calibration prior to initiation of the clinical service. If the principal professionals responsible for the SRS‐SBRT service do not have direct prior experience with the services to be offered, the facility must arrange for on‐site review and proctoring of the first clinical procedure by professionals with experience relevant to the new service.

## CONFLICT OF INTEREST

All members of Medical Physics Practice Guideline 9 completed disclosure statements and pertinent disclosures are shown below. These disclosures were reviewed by the Chair of the AAPM Subcommittee on Practice Guidelines and were determined to be sufficiently managed by disclosure and no other remedial measures were considered necessary.

Per Halvorsen: honoraria and travel expenses from ACR Radiation Oncology Practice Accreditation program; Eileen Cirino: none; Indra J. Das: none; Jeffrey A. Garrett: none; Jun Yang: none; Fang‐Fang Yin: none; Lynne A. Fairobent: none.

## Supporting information

Appendix A: SAMPLE Standard Operating Procedure (SOP) DOCUMENT
Appendix B: Example Staffing Policy

## NOTES

1 SRS and SRT using helical tomotherapy were excluded from the scope of this document due to the infrequent use of this technology for SRS and SRT. Exclusion from the scope of this document does not imply any AAPM position regarding the appropriateness of delivering such treatments using helical tomotherapy.

2 All treatments must occur under supervision of a QMP. In addition, a QMP must provide personal supervision at the first treatment, and as needed for subsequent treatments. The personal supervision should include participation in a time‐out checklist, assessment of patient immobilization, assessment of adequate imaging parameters, accuracy of respiratory management (if applicable), consultation on excessive or unusual patient shift requirements during treatment not clearly caused by patient motion on the treatment couch, as well as other patient‐ or plan‐specific needs.
